# The Effectiveness of Mindfulness-Based Cognitive Therapy (MBCT) in Real-World Healthcare Services

**DOI:** 10.1007/s12671-018-1087-9

**Published:** 2019-01-12

**Authors:** Alice Tickell, Susan Ball, Paul Bernard, Willem Kuyken, Robert Marx, Stuart Pack, Clara Strauss, Tim Sweeney, Catherine Crane

**Affiliations:** 1 Department of Psychiatry, University of Oxford, Oxford, UK; 2 NIHR CLAHRC South West Peninsula (PenCLAHRC), University of Exeter Medical School, Exeter, UK; 3 Tees Esk and Wear Valleys NHS Foundation Trust, Durham, UK; 4 Sussex Partnership NHS Foundation Trust, Sussex, UK; 5 Oxleas NHS Foundation Trust, Dartford, UK; 6 Sussex Partnership NHS Foundation Trust & School of Psychology, University of Sussex, Sussex, UK; 7 Nottinghamshire Healthcare NHS Foundation Trust, Nottingham, UK

**Keywords:** Mindfulness-based cognitive therapy, Depression, Implementation, Service delivery, Effectiveness

## Abstract

Depression is common with a high risk of relapse/recurrence. There is evidence from multiple randomised controlled trials (RCTs) demonstrating the efficacy of mindfulness-based cognitive therapy (MBCT) for the prevention of depressive relapse/recurrence, and it is included in several national clinical guidelines for this purpose. However, little is known about whether MBCT is being delivered safely and effectively in real-world healthcare settings. In the present study, five mental health services from a range of regions in the UK contributed data (*n* = 1554) to examine the impact of MBCT on depression outcomes. Less than half the sample (*n* = 726, 47%) entered with Patient Health Questionnaire (PHQ-9) scores in the non-depressed range, the group for whom MBCT was originally intended. Of this group, 96% sustained their recovery (remained in the non-depressed range) across the treatment period. There was also a significant reduction in residual symptoms, consistent with a reduced risk of depressive relapse. The rest of the sample (*n* = 828, 53%) entered treatment with PHQ-9 scores in the depressed range. For this group, 45% recovered (PHQ-9 score entered the non-depressed range), and overall, there was a significant reduction in depression severity from pre-treatment to post-treatment. For both subgroups, the rate of reliable deterioration (3%) was comparable to other psychotherapeutic interventions delivered in similar settings. We conclude that MBCT is being delivered effectively and safely in routine clinical settings, although its use has broadened from its original target population to include people experiencing current depression. Implications for implementation are discussed.

**Electronic supplementary material:**

The online version of this article (10.1007/s12671-018-1087-9) contains supplementary material, which is available to authorized users.

Depression is a common and debilitating mental health condition. Globally, it is thought to affect more than 300 million people and is a leading cause of disability-adjusted life years (WHO 2008). Of those people who access treatment, pharmacological and psychological approaches are most commonly used and are relatively effective in supporting people to remission (Hollon 2016; McManus et al. 2016). However, even when acute treatment is successful, people with a history of depression have a high risk of relapse/recurrence that increases with each successive episode: the likelihood is at least 40% after a first episode, 60% after a second, and as high as 90% after a third (Eaton et al. 2008; Moffitt et al. 2010; Solomon et al. 2000). Therefore, the clinical management of depression encompasses both acute and maintenance treatments.

Mindfulness-based cognitive therapy (MBCT) was developed as a relapse prevention programme, to help people who are at high risk of depressive relapse/recurrence to learn the skills to stay well in the long term (Segal et al. 2002). It is a psychosocial group-based intervention that comprises training in mindfulness meditation and elements of cognitive-behavioural therapy (CBT). There is evidence from at least nine clinical trials (*n* = 1258) that MBCT reduces the risk of relapse to depression when added to usual care, and demonstrates comparable efficacy to maintenance antidepressant medication (Kuyken et al. 2016). Studies comparing MBCT to closely matched psychological treatments have suggested comparable but not superior efficacy for relapse prevention (Farb et al. 2018; Manicavasgar et al. 2011; Meadows et al. 2014; Shallcross et al. 2015), over a period of up to 26-month follow-up (Shallcross et al. 2018). As such, MBCT is included in the clinical guidelines as a recommended option for relapse prevention in a number of different countries, including the UK, Netherlands, Canada, Australia and New Zealand (Malhi et al. 2015; NICE 2009; Parikh et al. 2016), and has been endorsed by the American Psychiatric Association (Lu 2015).

In line with these recommendations, many healthcare services are exploring how to include MBCT in community and public healthcare contexts, as part of the care pathway for people with recurrent depression. However, real-world mental health services are normally commissioned to address the needs of patients experiencing acute difficulties, whereas MBCT was developed for those who have remitted but are at risk of depressive relapse/recurrence. This has been a barrier to the implementation of MBCT in its original form, as people in remission are less likely to access services than those who are experiencing current problems. Some services have responded by adapting MBCT to fit their service delivery models (Crane and Kuyken 2013; Rycroft-Malone et al. 2017). For instance, there has been a move to widen the reach of MBCT to include people experiencing current depression (Strauss et al. 2014). There were initial concerns that MBCT may not be appropriate for this group, because practising mindfulness involves processes that could be difficult for those experiencing an acute depressive episode (e.g. sustained attention, bringing awareness to unpleasant feelings; Strauss et al. 2014). However, meta-analyses have demonstrated the efficacy of MBCT for reducing depression symptoms in patients with current depression (Hofmann et al. 2010). MBCT has been shown to perform as well as other comparable evidence-based treatments such as group CBT (Goldberg et al. 2018; Strauss et al. 2014). These effects appear to be maintained at follow-up, and robust when accounting for publication bias, study quality features and sensitivity analysis (Goldberg et al. 2018). Therefore, MBCT shows promise as an alternative psychological treatment for acute depression.

MBCT is thus increasingly considered suitable for patients with recurrent depression irrespective of their illness stage: in episode, in partial remission, or in full remission but vulnerable to relapse/recurrence. However, this evidence base reflects a relatively small number of studies, the majority of which are clinical trials in research settings. A recent review (Dimidjian and Segal 2015) mapped existing mindfulness research into its translational stages: basic science, intervention development and pilot testing, efficacy trials (in research settings), effectiveness trials (in community settings), and implementation. They highlighted that little research is being conducted in the later stages of the translational journey, namely effectiveness in real-world healthcare settings. In a research setting, MBCT is typically delivered to relatively homogeneous patient groups in accordance with strict research protocols and with a high degree of fidelity to manualised procedures. Conversely, community healthcare providers face real-world practical constraints and limited resources, which means they adapt treatment manuals to their service needs or broaden the population to which the intervention is applied (Onken et al. 2014). Such challenges can reduce a treatment’s effectiveness: for example, because key elements are unwittingly removed, it is delivered by therapists with less rigorous training, or administered to individuals with clinical presentations or sociodemographic characteristics that differ substantially from those for which the intervention was developed or in which its efficacy was tested (Henggeler 2011; Perepletchikova et al. 2007). In line with this, effectiveness studies of psychological interventions do typically show some decreases in intervention potency when a treatment is translated from research settings into the community (Curtis et al. 2004; Henggeler 2004; Miller 2005). However, it seems that diminishing effect sizes may not be inevitable. Some adaptations may be the result of ‘positive infidelity’: the introduction of well-informed modifications to an intervention’s delivery to ensure that it best meets the needs of the population to which it is being applied. Therefore, effectiveness research has important implications, highlighting whether more work is needed to adapt an intervention to suit the needs of real-world healthcare services, or whether it can be implemented in its existing format (Demarzo et al. 2015; Dimidjian and Segal 2015). This is critical to fulfil the public health goal of producing treatments that are both effective and implementable (Onken et al. 2014).

The primary aim of this study was to examine the effectiveness of MBCT for depression when offered in real-world healthcare settings. We used England as an exemplar, as it is currently one of the countries which has progressed furthest in terms of the formal implementation of MBCT in an integrated National Health Service (NHS), and providers routinely monitor the clinical outcomes of patients before and after treatment (Clark 2018). Existing clinical data were obtained from five healthcare services. These services offered MBCT to mixed groups of people with a history of depression: those in remission but at risk of depressive relapse/recurrence and those currently experiencing depression. It can be reasonably assumed that the target of treatment was different for each of these groups, and therefore, we examined separate outcomes for each. For service users entering treatment with depression symptoms in the non-depressed range, our question was whether MBCT sustained recovery and reduced risk of relapse. Service users were not followed up beyond the end of treatment, so in this subgroup, we conceptualised residual depression symptoms as a marker for risk of depressive relapse, based on previous studies which show that residual symptoms in the non-depressed range are a strong predictor of time to relapse/recurrence, even over long-term follow-ups (Ali et al. 2017; Judd et al. 1997; Pintor et al. 2004). For service users entering treatment with depression symptoms in the clinical range, our question was whether MBCT reduced the severity of depression symptoms and led to recovery. We also examined depression outcomes as a function of demographic characteristics, and in line with Kuyken et al. (2016), we predicted that outcomes would be similar for service users irrespective of their age and gender. Finally, because MBCT provision differed between the services on a number of dimensions, including the participant inclusion/exclusion criteria, staff training resources and requirements, and the provision of a full day of mindfulness practice, we conducted exploratory analyses to compare depression outcomes at each service.

## Method

### Participants

Five NHS services contributed data from 1554 service users who had each taken part in a group-based, face-to-face MBCT programme for adults (18+ years). The sample had a mean age of 49.37 years (*SD* = 12.74). Seventy-one percent of the sample were female, 89% were White British and 59% were employed. According to scores on the Patient Health Questionnaire (PHQ-9), before treatment, 53% of service users were currently depressed and 47% were not depressed. Table 1 shows the participant characteristics of the overall sample, each service, and subdivided into groups of services users who were currently depressed and non-depressed at entry to treatment.

**Table 1:** Baseline characteristics and attendance information for the pooled sample and each service, subdivided into non-depressed (‘No dep’) and depressed (‘Current dep’) at entry to treatment

Sample	*n*	Female %	White British %	Employed %	Age		Attendance		Dropout %^b^
					*M*	*SD*	*M*^a^	*SD*	
Pooled	1554	70.80^c^	88.85^c^	59.00^c^	49.37^c^	12.74^c^	6.37	2.39	16.93^c^
No dep	726	72.81^c^	90.23^c^	64.02^c^	49.68^c^	13.02^c^	6.42	2.35	16.26^c^
Current dep	828	69.10^c^	87.36^c^	52.27^c^	49.10^c^	12.50^c^	6.33	2.42	17.55^c^
Swallow	150	72.00	76.00	53.02^c^	44.92	12.09	5.25/8	2.06	20.00
No dep	78	71.79	83.33	51.95^c^	43.73	11.77	5.06/8	2.20	24.36
Current dep	72	72.22	68.01	54.17	46.21	12.39	5.44/8	1.90	15.27
Robin	508	70.47^c^	89.78^c^	–	49.01^c^	11.97^c^	5.77/8	2.32	20.28^c^
No dep	245	71.55^c^	89.06^c^	–	49.65^c^	12.36^c^	5.91/8	2.20	17.83^c^
Current dep	263	69.50^c^	90.43^c^	–	48.44^c^	11.60^c^	5.65/8	2.43	22.48^c^
Jackdaw	475	70.99^c^	93.24^c^	60.90^c^	50.92	12.95	6.92/9	2.31	14.11
No dep	280	74.05^c^	92.21^c^	67.39^c^	51.23	13.38	6.96/9	2.30	13.93
Current dep	195	66.84^c^	96.10^c^	51.56^c^	50.49	12.32	6.86/9	2.33	14.36
Woodpecker	181	67.40	–	–	47.45^c^	13.58^c^	–	–	–
No dep	59	74.57	–	–	48.56	13.57	–	–	–
Current dep	122	63.93	–	–	46.91^c^	13.61^c^	–	–	–
Blackbird	240	72.92	–	–	51.25	12.78	7.20/9	2.27	13.75
No dep	64	71.88	–	–	51.28	12.89	7.48/9	2.10	10.94
Current dep	176	73.30	–	–	51.24	12.77	7.10/9	2.32	14.77

Symbol ‘–’ denotes that no data was obtained

^a^Each service offered a different number of MBCT sessions, so at each service the mean number of sessions attended is presented against the total number offered

^b^Dropout refers to the percentage of participants who attended fewer than four MBCT sessions

^c^Due to missing data, the sample used to calculate the value in this cell differs from *n*. Refer to Supplementary Table S1 for the specific sample size

### Procedure

#### Recruitment

We recruited services that offered MBCT in line with the manualised programme in content and length (Segal et al. 2002) and routinely collected the PHQ-9 from service users at pre- and post-treatment. Of the MBCT services approached, five were eligible to participate, which covered four geographical regions in England and included a range of different types of service. MBCT services took part on the basis that they would be anonymised, so were given the following pseudonyms: Swallow, Robin, Jackdaw, Woodpecker and Blackbird. Swallow, Robin and Jackdaw were primary care services, part of a large-scale programme to make evidence-based psychological treatments widely available for people with common mental health problems, known as the Improving Access to Psychological Therapies (IAPT) Programme (Clark et al. 2009; Clark 2018); Woodpecker was a secondary care service, and Blackbird was a mixed primary and secondary care service. A full description of each service can be found in the Supplementary Materials, including the nature of MBCT provision and the service’s specific participant inclusion and exclusion characteristics. Table 2 provides a summary of the regional characteristics of each service, with information relating to the quality of NHS services, ethnicity, deprivation and prevalence of depression in the adult population.

**Table 2:** Summary of participating MBCT services with their regional characteristics

Pseudonym	Region in England	Service type	Quality rating^a^	White British %^b^	Deprivation index^c^	Depression prevalence %^d^
Swallow	London	IAPT	Good	60.3	129	7.2
Robin	East Midlands	IAPT	Good	85.0	115	8.9
Jackdaw	South East	IAPT	Good	88.4	137	10.3
Woodpecker	South East	Secondary care	Good	88.4	137	10.3
Blackbird	North East	Primary and secondary care (mixed)	Good	94.1	102	9.8

Each service belonged to an NHS foundation trust: an organisational unit within NHS England providing healthcare services to a particular geographical area. IAPT = Improving Access to Psychological Therapies programme (primary care)

^a^Quality rating refers to the rating given to the NHS foundation trust by the Care Quality Commission

^b^White British % was based on the local population obtained from Census data in 2011, averaged across the local authorities covered by the NHS foundation trust

^c^Deprivation index ranges from 1 to 209, where 1 = most deprived. This refers to latest indices of multiple deprivation (IMD) figures from 2015. The score was averaged across Clinical Commissioning Groups (CCGs) covered by the NHS foundation trust

^d^Depression prevalence % was calculated using the practice register aged 18+ in the 2016/17 Community Mental Health profiles, based on the Quality and Outcomes Framework, NHS Health and Social Care Information Centre (HSCIC). The score was averaged across CCGs covered by the NHS foundation trust

#### Design

The study used existing clinical data from NHS services. Services collected outcome measures as a part of routine clinical practice before and after treatment, although the timings differed according to the service: Swallow and Robin collected pre-treatment PHQ-9 scores from the total sample before the course started or in the first session. PHQ-9 scores were then collected again in each of the eight MBCT sessions. For the purposes of the present study, baseline and post-treatment data were provided, where post-treatment refers to the participant’s last measurement, not necessarily taken in the final session. As such, the time interval between baseline and post-treatment varied for each service user depending on their pattern of attendance at treatment. There was no information on pattern of session attendance available, so it was not possible to calculate the length of the interval between first and last data collection point. Jackdaw, Woodpecker and Blackbird collected pre-treatment PHQ-9 scores either before the course started or in the first session. PHQ-9 scores were collected again in the final session offered, at approximately an 8-week interval from the pre-treatment measurement. Service representatives accessed and anonymised the data to send to the investigators for analysis.

### Measures

#### Sociodemographic Information

Sociodemographic measures were used to determine the baseline characteristics of the sample. Each service provided data on the age and gender of service users. Some services had collected additional information on ethnicity and employment status, which was reported where available.

#### Session Attendance

All but one of the services (Woodpecker) provided information on the total number of MBCT sessions attended by service users. All services offered the core eight sessions (Segal et al. 2002). Some services also offered a pre-treatment orientation session or full day of mindfulness practice. Further information can be found in the Case Descriptions of each service (see Supplementary Materials).

#### Patient Health Questionnaire for Depression

The PHQ-9 was the primary outcome measure. It is a screening tool designed to establish a diagnosis of major depression and grade symptom severity (Kroenke et al. 2001). The nine-item measure corresponds to the nine symptoms of depression identified in the Diagnostic and Statistical Manual of Mental Disorders, 4th edition (American Psychiatric Association 2000). It is self-administered by the patient, and each item is rated on a scale of 0–3, yielding a score of depression severity between 0 and 27. A cutoff score of 10 or above indicates clinically significant depression symptoms, or ‘caseness’ (Kroenke et al. 2001). The PHQ-9 has demonstrated high internal reliability with Cronbach’s α ranging from 0.86 to 0.89 and high test–retest reliability (0.84; Kroenke et al. 2001). It has been validated in different patient groups and the general population (Kroenke et al. 2001; Martin et al. 2006; Spitzer et al. 1999; Spitzer et al. 2000). The factor structure of the PHQ-9 has been investigated in a number of different populations: in severe depression, it had two factors (‘affective’, e.g. depressed mood, feelings of worthlessness; ‘somatic’, e.g. sleep difficulties, appetite changes), which were stable over time up to 12 months (Guo et al. 2017).

### Data Analyses

#### Data Cleaning

Before analysis, the data were cleaned. First, missing data were managed. The reasons for missing data were administrative oversight or service users missing the relevant sessions. There were variable amounts of missing sociodemographic data across services and variables. In these cases, summary statistics were calculated using the data available. Details of the relevant sample sizes are provided in Table S1 in the Supplementary Materials. With respect to PHQ-9 scores, those with missing pre-treatment data were excluded from the analysis, as this information was crucial for subdividing the sample into those entering treatment above and below clinical cutoffs. Missing post-treatment data were handled using the last observation carried forward (LOCF) method. This provides the most conservative way of dealing with missing data in relation to the question of symptom reduction, the primary focus of this study, as it assumes no change over time. However, it should be noted that it would over-inflate estimates of sustained recovery, particularly if data were not missing at random but rather patterned by initial response to MBCT. The overall percentage of missing post-treatment data was 17.37%. Across the different services, the percentage of missing post-treatment data was as follows: Swallow (0%), Robin (0.79%), Jackdaw (26.95%), Woodpecker (40.33%) and Blackbird (27.08%). The impact of carrying forward the pre-treatment data therefore varied across the services depending on the amount of missing post-treatment data and the timing of assessments, and estimates of sustained recovery may most usefully be interpreted from Swallow and Robin.

The data were also checked for accuracy: out-of-range values were excluded as they resulted from errors in data entry. Data were checked for outliers, by inspection of the standardised z-scores for the difference between baseline PHQ-9 and post-treatment PHQ-9 values. Cases with z-scores in excess of 3.29 were treated as outliers. The analysis was run with and without these cases to examine their influence. Only six cases, distributed across four services, were identified as outliers. Omission of these values did not alter the pattern of results in any way. Findings for the full datasets are reported.

#### Effectiveness: Symptom Change

Change in the PHQ-9 from before-to-after MBCT was examined using paired *t* tests, with effect sizes quantified using Cohen’s *d*. When computing effect sizes, we did not correct for the correlation between the pre- and post-treatment means despite the within-subjects design (Morris and DeShon 2002); given that we used the LOCF method to deal with missing data, this would have artificially inflated the pre- to post-treatment mean correlations leading to less conservative estimates of effect size. Effect sizes were interpreted in line with Cohen’s (1988) criteria for effect sizes (small ≥ 0.20, medium ≥ 0.50 and large ≥ 0.80). This analysis was conducted for the pooled sample, each service, and for the subgroups of patients above and below the cutoff for current depression at entry to treatment. These subgroups were calculated based on the available clinical information: those scoring above the clinical cutoff (PHQ-9 ≥ 10) were classed as currently depressed, and those below (PHQ-9 < 10) were classed as without current depression (National Collaborating Centre for Mental Health 2018).

#### Effectiveness: Reliable Change

To benchmark the clinical relevance of the outcomes on the PHQ-9, the proportion of patients reporting a reliable change (either an improvement or a deterioration) at each service, and for each subgroup was calculated. A change between the first and last measurements is considered to be reliable if it exceeds the measurement error of the questionnaire. According to the guidelines laid down by the IAPT programme, which forms part of the UK NHS, the PHQ-9 has a reliable change index of ≥ 6 (National Collaborating Centre for Mental Health, 2018). Therefore, reliable improvement was said to have occurred if an individual had decreased in the PHQ-9 between pre- and post-treatment by 6 points or more, and reliable deterioration was said to have occurred if an individual had increased in the PHQ-9 between pre- and post-treatment by 6 points or more.

#### Effectiveness: Sustained Recovery and Recovery

For the subgroup who entered treatment with depression symptoms below the clinical cut-off (in recovery), we reported the number of patients who had sustained recovery following treatment. This index quantified the number of people who were below the clinical cutoff on the PHQ-9 before treatment and remained below the cutoff following treatment.

For the subgroup who entered treatment with clinical levels of depression, we also reported the number of patients who recovered following treatment. This index quantified the number of people that were above the clinical cutoff on the PHQ-9 before treatment but were below the cutoff following treatment. Finally, we also reported the rate of reliable recovery, which is said to have occurred if a patient above the threshold for depression at entry to treatment, moves below the threshold after treatment and also experiences a reduction in PHQ-9 score of 6 points or more.

#### Effects of Age, Gender and Service on Symptom Change

To examine whether the change in symptoms from pre- to post-treatment differed with participant age, linear regression analyses were conducted of the PHQ-9 change scores (outcome) on age (treated as a continuous variable). A *t* test was used to compare PHQ-9 change scores between male and female service users. There were insufficient data to analyse the sample based on the characteristics of employment status or ethnicity. A between-subjects ANOVA was conducted of the PHQ-9 change scores to test whether the change in PHQ-9 from pre to post treatment differed between services.

## Results

### Session Attendance

On average, the sample attended 6.37 sessions of MBCT (*SD* = 2.39) and 16.93% dropped out of treatment (attended less than 4 sessions). Table 1 shows the session attendance of the overall sample, each service, and subdivided into groups of services users who were currently depressed and non-depressed at entry to treatment.

### Effectiveness: Symptom Change, Reliable Change, Sustained Recovery and Recovery

In the pooled sample, comprising all service users irrespective of whether they were above or below the clinical cutoff on the PHQ-9 at entry to treatment, mean PHQ-9 score was *M* = 10.75 (*SD* = 6.18) at pre-treatment, and reduced to *M* = 7.81 (*SD* = 6.08) at post-treatment, resulting in a statistically significant change with a small to medium effect size, *t*(1553) = 22.46, *p* < 0.001, *d* = 0.48. In this pooled sample, 25.16% of service users experienced a reliable improvement in depression symptoms from pre- to post-treatment and 3.22% experienced a reliable deterioration.

Of the individuals who entered treatment below the clinical cutoff for depression, *n* = 726, 95.73% remained in the non-clinical range (showed sustained recovery) across the treatment period. There was a small further improvement in residual depression symptoms in this group over the treatment period, *t*(725) = 8.21, *p* < 0.001, *d* = 0.33, with 7.58% of individuals showing further reliable improvement and 4.13% showing reliable deterioration in symptoms.

Of those individuals entering treatment with depression scores above the clinical cutoff, *n* = 828, 40.58% showed a reliable improvement in depression symptoms over the treatment period and 2.42% showed a reliable deterioration in symptoms. In this group, 44.81% were recovered, and 34.42% were reliably recovered post-treatment and the pre- to post-treatment effect size for depression symptoms in this group was statistically significant and large, *t*(827) = 22.78, *p* < 0.001, *d* = 0.86.

Mean pre-treatment and post-treatment depression symptoms on the PHQ-9 are shown in Table 3, for the overall sample and subdivided according to level of depression symptoms at entry. Data on clinical indicators of change are shown in Table 4, for the overall sample and according to patients’ level of depressive symptoms at entry.

**Table 3:** PHQ-9 scores at pre- and post-treatment for the pooled sample and each service, subdivided into non-depressed (‘No dep’) and depressed (‘Current dep’) at entry to treatment

Sample	Pre-treatment		Post-treatment		Mean difference	95% CI	Cohen’s d
	*M*	*SD*	*M*	*SD*			
Pooled	10.75	6.18	7.81	6.08	2.94***	[2.69, 3.20]	0.48
No Dep	5.36	2.54	4.38	3.32	0.98***	[0.75, 1.22]	0.33
Current Dep	15.47	4.29	10.81	6.36	4.66***	[4.26, 5.06]	0.86
Swallow^a^	9.87	6.10	8.37	6.19	1.5**	[0.63, 2.37]	0.24
No Dep	4.92	2.81	5.19	3.75	− 0.27	[− 1.09, 0.56]	− 0.08
Current Dep	15.24	3.65	11.82	6.49	3.42***	[1.95, 4.89]	0.65
Robin^a^	10.56	6.23	6.83	5.79	3.73***	[3.21, 4.25]	0.62
No Dep	5.30	2.42	4.33	3.80	0.97***	[0.48, 1.45]	0.31
Current Dep	15.47	4.44	9.16	6.34	6.30***	[5.53, 7.08]	1.16
Jackdaw^a^	9.15	5.52	6.88	5.51	2.27***	[1.93, 2.61]	0.41
No Dep	5.37	2.58	3.95	2.74	1.43***	[1.15, 1.70]	0.53
Current Dep	14.56	3.86	11.08	5.76	3.48***	[2.79, 4.17]	0.71
Woodpecker^a^	12.84	6.49	10.87	6.64	1.96***	[1.34, 2.59]	0.30
No Dep	5.36	2.54	5.07	3.30	0.29	[− 0.51, 1.08]	0.10
Current Dep	16.45	4.36	13.68	6.00	2.78***	[1.96, 3.59]	0.53
Blackbird^a^	13.28	5.95	9.03	6.26	4.25***	[3.52, 4.98]	0.70
No Dep	6.09	2.38	4.83	2.87	1.27**	[0.47, 2.06]	0.48
Current Dep	15.89	4.53	10.56	6.46	5.34***	[4.43, 6.24]	0.96

Where post-treatment PHQ-9 data were missing, the pre-treatment PHQ-9 value was carried forward

^a^Pairwise comparisons were used to compare the pre- to post-treatment change in PHQ-9 scores between services (using Tukey HSD adjustment). There were differences between Robin and Swallow, Jackdaw, and Woodpecker, as well as between Blackbird and Swallow, Jackdaw and Woodpecker. In summary, Robin and Blackbird did not differ from one another in PHQ-9 reduction across their whole samples, and in both cases showed significantly larger pre-post treatment change than Swallow, Jackdaw and Woodpecker

**p < .01; ***p < .001

**Table 4:** Clinical indicators of change for the pooled sample and each service, subdivided into non-depressed (‘No dep’) and depressed (‘Current dep’) at entry to treatment

Sample	Reliable improvement %	Reliable deterioration %	Recovery %	Reliable recovery %	Sustained recovery %
Pooled	25.16	3.22			
No Dep	7.58	4.13			95.73
Current Dep	40.58	2.42	44.81	34.42	
Swallow	16.00	10.00			
No Dep	5.13	11.54			91.03
Current Dep	27.78	8.33	43.06	27.78	
Robin	34.06	4.72			
No Dep	9.39	6.12			93.88
Current Dep	57.03	3.42	59.70	48.29	
Jackdaw	17.26	0.63			
No Dep	6.79	0.36			98.93
Current Dep	32.31	1.03	36.92	27.18	
Woodpecker	18.23	2.76			
No Dep	3.39	5.08			93.22
Current Dep	25.41	1.64	22.13	17.21	
Blackbird	32.92	1.25			
No Dep	10.94	3.13			96.88
Current Dep	40.91	0.57	47.73	36.36	

Where post-treatment PHQ-9 data were missing, the pre-treatment PHQ-9 value was carried forward

### Effects of Age, Gender and Service on Symptom Change

Linear regression analysis showed no statistically significant effect of age (*p* = 0.09) on change in PHQ-9 score, and a *t* test showed no evidence of a difference in PHQ-9 change scores between genders (*p* = 0.7). A between-subjects ANOVA revealed a significant effect of service on change in PHQ-9 scores, *F*(4,1553) = 13.80, *p* < 0.001. Mean pre-treatment and post-treatment depression symptoms on the PHQ-9 are presented separately for each service in Table 3, and within each service according to patients’ level of depressive symptoms at entry. Across the individual services, those service users who were above the clinical cutoff for depression at entry showed greater pre- to post-treatment reductions in depression scores than those below the clinical cutoff, reflecting the same pattern of results as the pooled sample, and the smaller potential for further positive change in service users who were already below threshold on the PHQ-9 at treatment entry.

## Discussion

Although there are an increasing number of clinical trials examining the efficacy of MBCT for relapse prevention in recurrent depression and emerging evidence for its efficacy in treating acute depressive symptoms, little is known about the outcomes of MBCT when delivered in real-world healthcare settings, rather than research clinics (Dimidjian & Segal., 2015). In the present study, data were pooled from five clinical services within the UK NHS to examine patient outcomes. The services delivering MBCT were situated in different geographical locations, had different models of service delivery, and reached a relatively mixed sample of patients with respect to presenting problems (acute or recurrent depression or anxiety disorders), and sociodemographic characteristics, reflecting the range of service contexts in which MBCT is delivered ‘on the ground’. Thus, this study enabled us to examine the real-world outcomes of MBCT on depressive outcomes, for those in remission and for those experiencing current depression, and to compare outcomes as a function of gender and age. In the following sections we discuss the overall findings, their implications for ongoing provision of MBCT within healthcare settings and future research directions.

Overall, examination of the pooled data provided encouraging evidence of the acceptability of MBCT when delivered in the real world. Rates of session attendance were generally high and rates of drop out from treatment were relatively low and very similar to those observed in RCTs of MBCT for relapse prevention in recurrent depression (e.g. Kuyken et al. 2015; Ma and Teasdale 2004; Teasdale et al. 2000; Williams et al. 2014).

The fact that less than half the sample had depressive symptoms below the clinical cut-off on the PHQ-9 at entry to treatment suggests that in routine clinical practice the use of MBCT has broadened from its original intention as a relapse prevention intervention, to be used with patients who are symptomatic (see also Rycroft-Malone et al. 2017, which provides convergent evidence that services are adapting MBCT). We examined the outcomes of these two groups separately, on the premise that their treatment targets would be different. In the group who was below the clinical cutoff on the PHQ-9 at entry to treatment, the treatment target would be sustained recovery; 96% sustained recovery across the treatment period. Although the LOCF method of dealing with missing data may have inflated this proportion, the rate of sustained recovery at Robin, where there was no missing data, was 94%, suggesting that any such effect is unlikely to be marked. Furthermore, there was a significant reduction in residual depression symptoms, *d* = 0.33, despite there being a restricted potential for positive change. Many studies have demonstrated the clinical importance of reducing residual depression symptoms, as they have a large impact on long-term depression outcomes (Rottenberg et al. 2018). In the absence of long-term follow-up of service users, a reduction in residual symptoms of depression can also be regarded an imperfect proxy for reduction in risk of relapse (Ali et al. 2017). Although a measure of relapse beyond the end of treatment would have been a more appropriate outcome measure for this subgroup, this information was not available from clinical services. However, the pre- to post-treatment effect size identified in the present study was comparable to that of a large RCT (*d* = 0.35; Kuyken et al. 2015), where the main outcome variable was time-to-relapse over 24 months, and outcomes were shown to be comparable between MBCT and maintenance antidepressant medication. This suggests that MBCT in the real world may produce benefits comparable to those found in RCTs in terms of supporting recovery, at least over the limited follow-up periods.

In the group who entered MBCT with depression (e.g. above cutoff on the PHQ-9 for caseness), results showed large and significant improvements in depressive symptoms, *d* = 0.86, and a 34% rate of reliable recovery. In the absence of a randomised design with a no-intervention control group, it is not possible to determine the extent to which these improvements in depressive symptoms are attributable to natural recovery rather than intervention effects. However, it should be noted that the effect sizes observed are similar to those reported in a study that documented pre- to post-intervention changes in depressive symptoms in patients randomised to MBCT or treatment as usual (van Aalderen et al. 2012). Where substantially larger effects have been observed, these have been based on small samples (Barnhofer et al. 2009; Chiesa et al. 2015; Eisendrath et al. 2015; Kingston et al. 2007). In the present study, the effect on PHQ-9 symptoms was smaller compared to interventions offered in IAPT services overall for depression cases (1.4; Clark 2018). However, the rate of reliable improvement (41%) was comparable to that of CBT in a sample of service users at 103 IAPT services that were above the clinical cutoff on the PHQ-9 at intake (47%; Pybis et al. 2017). Service users in the present study most commonly took part in MBCT as a second-line treatment after another form of psychological therapy (typically CBT) and had lower PHQ-9 scores at intake than the IAPT samples mentioned above. Therefore, one might expect a smaller effect size in this group compared to the IAPT sample that also included those who took part in CBT as a first-line treatment. Nonetheless, an important remaining question is, ‘What approach is most acceptable, effective, and cost-effective for different subpopulations of people with depression, at different stages in the natural history of depression (e.g. first episode, recurrent depression)?’

Finally, we asked if MBCT was equally helpful for people of different ages and genders. Consistent with Kuyken et al. (2016), neither age nor gender was associated with the degree of reduction in depressive symptoms during treatment, indicating that outcomes do not seem to be influenced by these sociodemographic variables.

### Interpretation of Service-Specific Outcomes

Our study examined data from five clinical services which differed to some degree in the populations served (in particular with respect to clinical eligibility), the intervention delivered (e.g. number of sessions and adaptations), and the structure of the services including the training pathway completed by MBCT instructors. There were also marked differences between services in the availability of post-treatment data. Whilst findings were broadly consistent across services, there were also areas of divergence, and the services differed significantly from one another clinical outcomes. Attributing these differences to particular service characteristics is challenging. The measured sociodemographic characteristics of service users and their clinical symptoms at baseline did not show marked differences between services, although it is possible that there were other differences not captured by routine data collection. It is also possible that differences between services are related to models of MBCT teacher training, supervision and service delivery. In the absence of data on teacher competency or intervention fidelity, this is speculation, but it nonetheless raises the important possibility that the way services offer MBCT can affect outcomes. Future research might evaluate outcomes over a larger number of clinical services, varying in patient eligibility requirements, MBCT teacher training pathways, and models of service delivery, to shed light on how patient and service factors interact to determine outcomes. In addition, an in-depth study of how adaptations in psychoeducational content or the issues explored in group enquiry in MBCT influence the learning of patients with different presenting symptoms and clinical histories might shed light on the best way to diversify services to broader populations without diluting treatment effects.

### Safety of MBCT in Real-World Settings

The fact that rates of reliable deterioration are below 5% for the total sample and for subgroups entering above and below clinical cutoff for depression, suggests that MBCT is a treatment option which is generally safe, and that reliable deteriorations occur no more frequently after treatment with MBCT than with other psychotherapies. For example, Hansen et al. (2002) report a reliable deterioration rate of 8.2% in a sample of more than 6000 individuals receiving various forms of psychotherapy in real-world settings in the USA, whilst Crawford et al. (2016) found that in a survey of over 14,000 UK NHS psychological therapy patients, just over 5% reported lasting bad effects of treatment. Similarly, the observed rates of reliable deterioration are comparable to those observed in IAPT services for anxiety and depression as a whole (e.g. Clark 2018). Results show that those entering MBCT treatment with depression symptoms below the clinical cutoff are still almost twice as likely to reliably improve further as to deteriorate over the course of treatment (although absolute proportions showing both types of change are low), even from their low level of baseline symptoms, and in the context of a high risk of relapse. Furthermore, of those entering treatment above the clinical cutoff for depression, the proportion who reliably improved was 17 times greater than the proportion who reliably deteriorated. These figures should be interpreted with some caution since where data were missing at follow-up, baseline data were carried forward. Thus, in the current analysis, for services where data were collected only at the first and final sessions of treatment, participants experiencing a significant worsening of symptoms and dropping out of treatment would be recorded as having no change in symptom levels whereas in fact symptoms might have worsened. Likewise, in such services levels of sustained recovery are likely to over-estimate real effects. Robin, the largest unique sample, with negligible missing data, had relatively low rates of reliable deterioration (4.7%), high rates of reliable improvement (34%) and high rates of sustained recovery (94%), whereas Jackdaw, in contrast, had very high rates of missing data (and hence baseline data carried forward) and showed reliable deterioration rates of 0.63%, reliable improvement rates of 17% and rates of sustained recovery of 99%. This pattern of results suggests that the approach we adopted of carrying forward baseline data where outcome data were missing data may be suppressing rates of both reliable improvement and deterioration, and exaggerating rates of sustained recovery. Robin, as the largest and most complete sample, probably provides the most robust estimate of reliable deterioration, reliable improvement and sustained recovery to be expected from MBCT in routine care settings.

### Accessibility

Overall, the rates of engagement suggested that those electing to start MBCT found it acceptable. However, despite the fact that MBCT services were located in relatively ethnically diverse communities, the participants attending treatment were predominantly White. For example, Swallow has a regional ethnicity of 60% White British, compared to 76% of those receiving MBCT and providing ethnicity data. Likewise, Jackdaw serves a population that is 88% White British, compared to 93% of those receiving MBCT and providing ethnicity data. It is well established that people from Black, Asian and minority ethnic backgrounds in the UK face barriers to accessing mental health services (e.g. Sashidharan 2003; Memon et al. 2016). It is not clear to what extent the disparities in ethnic composition in our datasets reflect broader structural barriers to accessing mental health services by people from these ethnic backgrounds, or whether there are particular issues with access to and acceptability of MBCT. Existing clinical trials of MBCT for relapse prevention in depression have also focussed largely on Caucasian samples and/or have not reported information on the ethnicity of participants (e.g. Goldberg et al. 2018; Kuyken et al. 2016). Whilst this aids the comparison of the results in this dataset with those of previous research, it highlights that issues of equality and diversity in access to MBCT are an area where future research and action is urgently needed.

### Strengths and Limitations

This study drew existing data from real-world clinical services. This is a strength of the work as it provides a naturalistic picture of the effectiveness of MBCT as it is delivered in healthcare settings, without any experimental interference or control. However, this also brought with it certain challenges. In particular, there were high rates of missing data in some services, which, as discussed above, may have influenced both estimates of positive and negative treatment effects. Likewise, data on variables such as service-users’ clinical history, additional ongoing treatments, instructor competence, class size and composition, and service user engagement with home practice, which might be hypothesised to influence outcomes, were not available. Similarly, we did not have access to information on the groupings of individuals receiving MBCT, so in our analysis, we were unable to correct for the intragroup correlations between participants. Previous studies of MBCT have not found significant intragroup dependency, and re-analysis of datasets using hierarchical linear modelling has been found to reinforce original findings (Williams et al. 2008). However, this may not be the case in real-world MBCT services, and so, we recommend that healthcare services could store information on class grouping so that this can be taken into account in future studies. Another limitation was that this study recruited services from NHS England, and so the findings only reflect this country’s model of service provision. Models of delivery in other countries are likely to differ from that of England, and so, it is unclear whether the outcomes of this study would translate to other forms of MBCT delivery taking place internationally.

In summary, our findings suggest that MBCT in real-world settings is acceptable and effective. However, it is important to reflect on the fact that MBCT was first designed as a relapse prevention treatment for individuals at high risk of depressive relapse to learn skills whilst well (i.e. with below threshold levels of depressive symptoms). In line with other recent work (Rycroft-Malone et al. 2017), services are offering MBCT to a broader population. In our sample just under 47% of service users entered treatment with levels of depressive symptoms below the clinical cutoff on the PHQ, and thus might be considered to have been entering MBCT for relapse prevention purposes. Encouragingly, in this subgroup, there were very high rates of sustained recovery, low rates of sustained deterioration a small and statistically significant effect on residual symptoms, which are consistently shown to be the best predictor of depressive relapse (Bockting et al. 2015).

However, 53% of our sample entered MBCT with acute depressive symptoms, and whilst acceptability and effectiveness were again encouraging, the effect size for outcomes, *d* = 0.86, whilst large, is smaller than for IAPT depression services as a whole (Clark 2018). Therefore, the current data suggest that whilst MBCT can be safely and effectively delivered to individuals who are both in remission and symptomatic, we would suggest it may be best conceptualised as a second-line treatment. Indeed, many services have chosen to offer MBCT in this way. This approach recognises that depression is typically a lifelong, recurrent disorder, and MBCT can add benefit after other treatments designed more specifically to address acute symptoms. In line with an agenda to increase patient choice, it provides an alternative approach for people wishing to better manage their depression. Indeed, further RCTs addressing issues of treatment sequencing, the acute and relapse–prevention effects of MBCT when offered irrespective of stage of illness, and long-term preventative effects of MBCT in real-world settings are now required. This work would be enriched by broadening outcomes beyond symptomatology and disorder status to include functional status, quality of life and living well with depression.

## Electronic supplementary material

ESM 1 (DOCX 23 kb)
