# Supplementary material for: The Effectiveness of Mindfulness-Based Cognitive Therapy (MBCT) in Real-World Healthcare Services
Source: Mindfulness (N Y). 2019 Jan 12;11(2):279–90. doi: 10.1007/s12671-018-1087-9 (PMC6995449; doi:10.1007/s12671-018-1087-9)
Supplement: Supplementary file 1 — (DOCX 23 kb) [file 12671_2018_1087_MOESM1_ESM.docx]

**Supplementary Materials**

**Case Descriptions**

Below is a summary of the participating MBCT services, including the nature of MBCT provision as well as participant inclusion and exclusion characteristics. Information was collected by one of the authors, using semi-structured interviews with the lead MBCT practitioner representing each service.

**Primary care services (IAPT)**

*Swallow.* Swallow was an IAPT service established in 2009. MBCT courses began in 2012, offered roughly four times per year. Six MBCT teachers were employed over the timescale of the dataset, together corresponding to 0.4 whole time equivalent staff. These staff were either dedicated MBCT teachers or other mental health professionals who had undergone five days of intensive MBCT training. The MBCT programme consisted of an orientation followed by eight weekly sessions. The service was provided for adults with mild symptoms of depression or anxiety. Previously, MBCT had been restricted to people presenting with depression as their primary problem, but the service had recently been extended to people experiencing anxiety disorders. The inclusion criteria were: a history of depression or anxiety, age 18 years or over, living or having a GP in the borough, and willingness to engage in a group intervention. Exclusion criteria were: high risk of suicide, dependency on alcohol and/or substances, primary diagnosis of a severe mental health problem (e.g. psychotic illness, personality disorder). Patients could self-refer to the service or be referred by their General Practitioner. Up to 15 people were invited to attend in each group, but on average 6-8 service users attended, with 1-2 teachers. Data were collected from service users over a six-year period from 2012 – 2017. The data included sociodemographic measures of age, gender, ethnicity, employment status, and number of sessions attended.

***Robin.*** Robin was an IAPT service providing psychological therapies to adults with mild-to-moderate, common mental health problems. MBCT courses began in 2010, offered at least five times per year. Ten MBCT teachers were employed at the time of interview, together forming one whole time equivalent post. The NHS trust had its own accredited ‘Mindfulness Centre’, where existing staff could follow a one-year, part-time training pathway to teach MBCT, with ongoing supervision and workshops. All MBCT teachers were qualified mental health professionals. The MBCT programme consisted of an orientation followed by eight weekly sessions. The MBCT service did not use strict inclusion or exclusion criteria, and clinical judgement was used to determine a person’s eligibility to participate. Generally, patients had recurrent depression and were either in remission or close to remission. Patients could self-refer to the service or be referred by their General Practitioner. Up to 15 people were invited to attend each group, with two teachers. Data were collected from service users over a seven year period from 2010 – 2017. The data included sociodemographic measures of age, gender, ethnicity, and number of sessions attended.

***Jackdaw.*** Jackdaw was an IAPT service established in 2009, offering a range of mental health support for adults with common mental health problems such as low mood, stress, anxiety and depression. MBCT courses began in 2010, offered at least nine times per year. At the time of interview there were three MBCT teachers, together corresponding to 1.5 whole time equivalent staff. The NHS trust had its own accredited Mindfulness Centre and a one-year training pathway with ongoing supervision and seminars. The MBCT programme consisted of an orientation followed by eight weekly sessions. The MBCT service did not have strict inclusion or exclusion criteria. Rather, clinical judgment was used to determine a person’s eligibility to participate. MBCT was offered to adults experiencing depression and/or anxiety, most commonly as a second-line treatment after another form of therapy. Generally, patients experiencing psychosis, trauma, or recent bereavement were excluded. Patients could self-refer to the service or be referred by their General Practitioner. Up to 15 people were invited to attend each group, with two teachers. Data were collected from service users over a seven-year period from 2010 – 2017. The data included sociodemographic measures of age, gender, employment, ethnicity, and number of sessions attended.

**Mixed primary/secondary and secondary care services**

***Woodpecker.*** Woodpecker was a secondary care service established in 2012 for patients needing specialist mental healthcare, offering 2-4 MBCT courses per year. Woodpecker belongs to the same NHS trust as Jackdaw, but Jackdaw and Woodpecker were distinct MBCT services offered to different patient populations. At the time of interview, the service had 12 MBCT teachers who were all mental health professionals (mostly clinical psychologists), together corresponding to 0.5 whole time equivalent staff. As with Jackdaw, the MBCT teachers had undergone a one-year supervised training pathway at the NHS trust’s Mindfulness Centre. The MBCT programme consisted of an orientation followed by eight weekly sessions. MBCT was offered on the basis of clinical judgement, to adults experiencing depression and/or anxiety, most commonly as a second-line treatment after another form of therapy. However, given that the service was for patients receiving secondary care, patients would typically be expected to have more severe and complex mental health problems those taking part in MBCT with Jackdaw. Up to 15 people were invited to attend each group, with two teachers. Data were collected from service users over a five-year period from 2012 – 2017. The data included sociodemographic measures of age and gender, but no information on the number of sessions attended.

***Blackbird.*** Blackbird was an MBCT service established in 2015, available to a mixed group of patients across both primary and secondary care, initially offering approximately 15 courses per year. At the time of interview, 6 MBCT teachers were employed, together corresponding to 3.6 whole time equivalent staff. MBCT teachers had undergone a variety of trainings, including a one-year supervised training pathway at the NHS trust’s own accredited Mindfulness Centre. The MBCT programme consisted of a two-hour taster session, followed by a telephone assessment, eight weekly sessions, and a full day of mindfulness practice. MBCT courses were offered to adults who had experienced recurrent depression, for symptom reduction as well as relapse prevention. There were no strict inclusion or exclusion criteria, but generally MBCT was offered to people with three or more previous episodes of depression, who were still eligible to participate even if they were currently experiencing significant depressive symptoms. The courses were also offered to stressed carers and parents of patients, and some of the courses were also open to NHS trust staff. Because the groups were mixed, minor adaptations were made to the original course materials to make them suitable for the range of people attending (i.e. taking out some depression-specific terminology). Data analysis for the present study was limited to the patients identified as having recurrent depression. Patients accessed the service through self-referral. The service was open to patients from both primary and secondary care settings. Up to 20 people were invited to attend in each group, but on average 12-15 service users attended, with usually just one teacher. Data were collected from service users over a two-year period from 2015 – 2017. The data included sociodemographic measures of age and gender, and the number of sessions attended.

| Sample | *n* | Female % | White British % | Employed % |  | Age | |  | Attendance | | Dropout % |
| --- | --- | --- | --- | --- | --- | --- | --- | --- | --- | --- | --- |
|  |  |  |  |  |  | *M* | *SD* |  | *M/Total* | *SD* |  |
| Pooled | 1554 | 1517 | 906 | 617 |  | 1536 | 1536 |  | 1353 | 1353 | 1353 |
| No Dep | 726 | 695 | 471 | 353 |  | 713 | 713 |  | 652 | 652 | 652 |
| Current Dep | 828 | 822 | 435 | 264 |  | 823 | 823 |  | 701 | 701 | 701 |
| Swallow | 150 | FS | FS | 149 |  | FS | FS |  | FS | FS | FS |
| No Dep | 78 | FS | FS | 77 |  | FS | FS |  | FS | FS | FS |
| Current Dep | 72 | FS | FS | FS |  | FS | FS |  | FS | FS | FS |
| Robin | 508 | 491 | 401 | ND |  | 491 | 491 |  | 488 | 488 | 488 |
| No Dep | 245 | 232 | 192 | ND |  | 232 | 232 |  | 230 | 230 | 230 |
| Current Dep | 263 | 259 | 209 | ND |  | 259 | 259 |  | 258 | 258 | 258 |
| Jackdaw | 475 | 455 | 355 | 468 |  | FS | FS |  | FS | FS | FS |
| No Dep | 280 | 262 | 201 | 276 |  | FS | FS |  | FS | FS | FS |
| Current Dep | 195 | 193 | 154 | 192 |  | FS | FS |  | FS | FS | FS |
| Woodpecker | 181 | FS | ND | ND |  | 180 | 180 |  | ND | ND | ND |
| No Dep | 59 | FS | ND | ND |  | FS | FS |  | ND | ND | ND |
| Current Dep | 122 | FS | ND | ND |  | 121 | 121 |  | ND | ND | ND |
| Blackbird | 240 | FS | ND | ND |  | FS | FS |  | FS | FS | FS |
| No Dep | 64 | FS | ND | ND |  | FS | FS |  | FS | FS | FS |
| Current Dep | 176 | FS | ND | ND |  | FS | FS |  | FS | FS | FS |

Table S1

*Sample sizes used to compute the value in each cell of Table 1: Baseline characteristics and attendance information for the pooled sample and each service, sub-divided into non-depressed (‘No dep’) and depressed (‘Current dep’) at entry to treatment.*

*Note*. FS = full sample; ND = no data.
